# 

**Published:** 1887-07

**Authors:** 


### OUR ADVERTISERS.

DR. J. J. SULLIVAN, Lowell, Mass., writes: "Colden's Liquid Beef Tonic" excels all other beef extracts now in use.

LACTOPEPTINE.—Read the new and attractive advertisement of this valuable digestive agent in this issue of THE JOURNAL.

A PERFECT LIQUID CATHARTIC.—Dr. Geo. W. Hoagland Columbus, Ohio, writes: "I can truly say that I have used 'Elixir Purgans (Lilly)' with great satisfaction in cases of obstinate constipation, and can cheerfully recommend the use of this preparation to other practitioners of medicine."

WE call the attention of our readers to the advertisement of Messrs. R. A. Robinson & Co., Louisville, Ky., which will be found on another page of this issue. This firm was established forty-five years ago, and enjoys a wide-spread reputation as a sound, honest, reliable business house. We do not hesitate to endorse their preparations as being all they claim for them.

MEDICAL COLLEGE ANNOUNCEMENTS.—We present quite a number of new advertisements of medical colleges in this issue to which we desire to direct the attention of those of our readers who are interested. Physicians having students who will enter college during the coming autumn will be able to find a college which will suit them from the list we present. Read the advertisements and write for announcements.

LONG ISLAND COLLEGE HOSPITAL.—The reading and recitation term of the Long Island College Hospital, which began in March, has just closed. During the three months' session the students have had the advantages of the new Maternity Wards of the Hospital, and have attended two abortions and seventeen confinements. In attendance upon these cases they witnessed the following complications and operations: Complete inversion of uterus and its reduction, 1; Placenta praevia, 2; Transverse presentations, 3; Twins, 1; Hydrorrhœa (post partum), 1; Forceps deliveries, 2; Breech, 2; Version, external, 2; Version, internal, 1; Restoration of perineum, 3; Curretting after abortion, 2.

BROMIDIA.—W. H. MAY, M. D., No. 20 W. Twenty-Fourth Street, New York city, says: I have had very successful results in the administration of Bromidia in cases having their origin in disorders of the nervous system, such as cholera infantum, paralysis, insomnia, etc. *But I find it to be of special value in treatment of delirium tremens, and the results of debauch*—it being retained upon the stomach and speedily controlling the most dangerous symptoms, and producing the desired calmness and sleep necessary when morphia and other soporifics have failed to do so, and thus rendering the disorder amenable to further treatment. Have also prescribed it successfully in the terrible state of nervous exhaustion due to opium habitues endeavoring to relinquish the habit. And, finally, as result of experience, I pronounce it the "hypnotic *par excellence*."

THE wide-awake firm of Seabury & Johnson (Seabury Pharmacal Laboratories), New York, have added to their list of laboratory products a full line of medicated and antiseptic soaps of a high degree of excellence. Conspicuous in this addition are their *one* and *five* per cent. hydronapthol soaps; the former for the toilet, the latter for medicinal and antiseptic purposes. These soaps are prepared from the pure ingredients obtainable, highly scented, entirely neutral, containing no free alkali, acid or impure fat. Dr. C. W. Allen, of New York, and other prominent skin specialists recommend these hydronapthol soaps very highly in the treatment of ulcers, eczema scabies, impetigo, pruritus, pityriasis capitis, alopecia, favus, etc., etc. Aside from their healing powers these soaps also impart their well-known powerful antiseptic and disinfectant properties to the waters in which they are used, and in this way free basins, bath-tubs, cesspools, sinks, etc., etc., from all unpleasant odors and foul gases.

